# In-vitro fertilization and spontaneous pregnancies: matching outcomes in Douala, Cameroon

**DOI:** 10.1186/s40738-015-0013-2

**Published:** 2016-01-19

**Authors:** Thomas Obinchemti Egbe, Guy Sandjon, Clovis Ourtchingh, André Simo, Eugene Belley Priso, Jean-Louis Benifla

**Affiliations:** 1 Faculty of Health Sciences, University of Buea; Department of Obstetrics and Gynecology, Douala General Hospital, Douala, Cameroon; 2 Clinique de l’Aéroport, Douala, Cameroon; 3 Department of Obstetrics and Gynecology, Regional Hospital Maroua, Douala, Cameroon; 4 Faculty of Medicine and Biomedical Sciences, University of Yaoundé and Department of Obstetrics and Gynecology, Douala General Hospital, Douala, Cameroon; 5 Service de Gynécologie-Obstétrique, Hôpitaux Universitaire Saint-Louis Lariboisière Fernand-Widal, 2 rue Ambroise Pare 75475, Paris, Cedex 10 France

**Keywords:** Cesarean delivery, In-Vitro Fertilization, Natural conception, Vaginal delivery

## Abstract

**Background:**

Couples are considered infertile if they do not conceive over a 12-month period of unprotected intercourse. Studies have shown that female causes accounted for between 25 to 37 percent of infertility worldwide (with larger proportions in sub-Saharan Africa and Southeast Asia) and male causes accounted for between 8 to 22 percent. Both male and female causes accounted for between 21 to 38 percent. Although the majority of ART children are normal, there are concerns about the increased risk for adverse pregnancy outcomes. More than 30 % of ART pregnancies are twins or higher-order multiple gestations (triplets or greater) and more than one half of all ART neonates are the products of multifetal gestations, with an attendant increase in prematurity complications. The aim of this study was to evaiuate the outcome of pregnancies conceived by In-vitro fertilisation compared to those conceived naturally in two hospitals in Douala, Cameroon.

**Methods:**

This was a prospective study carried out from October 1, 2011 to September 30, 2012. Participants were recruited from two hospitals: the Douala General Hospital (DGH) and the Clinique de l’ Aéroport (CDA), also in Douala. A total of 102 women were recruited for study: 51 who conceived by IVF (cases) and 51 who conceived naturally (controls). Of the 102 women, 52.9 % were between 31 – 39 years of age, while 21.6 % were above 40.

**Results:**

Participants who conceived through IVF-ET were 4.1 times more likely to undergo cesarean delivery than those who conceived naturally [OR 4.10, 95 % CI 1.78–9.42]. Similarly, a higher percentage of patients in the IVF group than those in the control group have never given birth (33.3 % vs 2.0 %) (*P* < 0.0001). The percentage of multiple pregnancies was 7.5 times higher in the IVF group than in the control group (14.7 % vs.1.96 %) (*P* = 0.000).

The leading indication for cesarean delivery was advanced maternal age (27.3 %) followed by IVF or precious pregnancy (18.2 %).

**Conclusions:**

Cesarean delivery was more frequent amongst the IVF group than in the control group. The leading indications for cesarean delivery were advanced maternal age and IVF or precious pregnancy.

The long-term neonatal outcomes of IVF babies beyond 5-min Apgar scores should be studied in Cameroon and follow-up beyond 1 year encouraged.

## Background

Since the introduction of In-Vitro Fertilization (IVF) in 1978 [1], an estimated 3 million babies have been born worldwide through this procedure [2]. The first baby born after IVF was Louise Brown, by cesarean section, on July 25, 1978 at Oldham hospital in the United Kingdom (UK) [1]. The birth of Louise Brown opened a new era in the management of the infertile couple. Today, there are many developments in terms of pharmaceutical substances, management protocols and laboratory techniques that have completely changed the approach to the management of infertility. The resultant effect is an increase in the number of children born to mothers through assisted reproductive technology (ART) [3].

An estimated 2–3 % of children born in some Scandinavian countries, especially Denmark, are conceived through ART [4]. With the introduction of IVF, there has been a higher rate of cesarean delivery among IVF cases than among women who conceived naturally (41.9 versus 15.5) [5]. This trend can be explained, on the one hand, by the excessive cautiousness of the obstetricians, and on the other by stress on the part of the couples caused by such things as birth trauma or fear of losing the baby. ART pregnancies are more at risk of induction of labour and elective cesarean section than those in the control group [6, 7]. A similar trend in cesarean sections has been reported in Europe and the United States. This rate has remained high over the years, in keeping with the marked increase in the number of IVF centers worldwide [3].

In Cameroon, the practice of ART was introduced in 1998 [8]. Since then an unpublished number of babies have been born to IVF mothers. The aim of this study was to evaluate the outcome of pregnancies conceived by IVF compared to those conceived naturally.

## Materials and Methods

The study population included 51 consecutive IVF pregnancies obtained between October 1, 2011 and September 30, 2012 at the Clinique de l’ Aeroport (CDA) in Douala Cameroon, and from other centers out of Cameroon. All the patients were accepted for IVF because this was the only way they could obtain a pregnancy. The indication for IVF was tubal obstruction in all the cases. In Cameroon, IVF does not enjoy medical insurance coverage and so is not available to all socio-economic classes. The analysis included only pregnancies leading to a live birth (>28 weeks gestation or >1000 g birth weight).

Throughout the period of study, the same team of specialists worked in the IVF unit of CDA and treatment protocols remained the same. Controlled Ovarian Hyperstimulation (COH) in all patients was done with human menopausal gonadotropin (hMG) after desensitization with triptorelin (GnRH-a) initiated in the luteal phase (day 21) of the cycle. Human chorionic gonadotropin (hCG) was administered when the leading follicle reached 17–18 mm in diameter as measured by transvaginal sonography and serum E2 levels >500 pg/mL. Standard IVF was used in all cycles. We usually transferred 3 fresh embryos and did not perform micromanipulation in any of the cycles. Our luteal phase support was with progesterone pessaries 200 mg, thrice daily, from the day of embryo transfer to the day of the pregnancy test.

The antenatal care of the patients was performed at CDA and the Douala General Hospital (DGH), and all the patients gave birth in these two centers. Complete data regarding the course and outcome of these pregnancies were available in CDA and the DGH.

The control group consisted of 51 spontaneous gestations that were delivered at the DGH and CDA and were therefore treated by the same obstetric department as the cases. The control for each index pregnancy was the consecutive delivery at CDA and DGH matched for maternal age and with similar expected date of delivery (calculated from the first day of the last normal menstrual period).

The exclusion criteria were women with previous IVF or spontaneous pregnancies who delivered by cesarean section and/or delivered before 28 completed weeks of gestation.

### Data collection

The records of the participants were reviewed and the data recorded on standardized survey questionnaires. These data were obtained from the IVF unit files, antenatal care records, and maternal and neonatal delivery and hospitalization records. Data on patients who did IVF in centers out of Cameroon were obtained from the patients.

The data focused essentially on medical and obstetric history, investigations, cause of infertility, pregnancy course and antenatal complications if any, course and mode of delivery, complications during labour and the puerperum, status of the infant at birth, and admissions into the neonatal intensive care unit. In the study group, gestational age was calculated as if the first day of the last menstrual period had been 14 days before the day of oocyte retrieval. Gestational age of the controls was confirmed by routine ultrasound scanning. The variables obtained were then controlled for both groups.

### Statistical analysis

Epi-info 6.04 and R software were used for statistical analysis. The chi-squared test was used to compare rates of cesarean and vaginal delivery according to the following characteristics: technique of conception, age range of patients, number of children alive, parity, duration of infertility, IVF center and number of trials, gestational age at delivery, delivery institution. A logistic regression analysis was used to compute Odds ratios (OR’s). Statistical significance was set at *P* <0.05.

### Ethical approval

Ethical clearance was obtained from the Institutional Review Board (IRB) of the Faculty of Health Sciences, University of Buea, Cameroon. Permission was granted by the Directors of the Douala General Hospital and the Clinique de l’Aeroport. Informed consent was obtained from participants.

## Results

### Maternal socio-demographic characteristics

The socio-demographic characteristics of 51 consecutive pregnancies obtained at the Clinique de l’Aeroport and IVF centers out of Cameroon were matched with those of 51 control spontaneous pregnancies for maternal age, education, marital status, religion and profession. The maternal characteristics of both groups are presented on Table 1. The age of the pregnant women ranged between 31–39. The characteristics studied were similar in both groups.

**Table 1:** Maternal Socio-demographic characteristics

Characteristics	Total		IVF-ET		Controls		*P-value*
	*N*=102	(%)	*N*=51	(%)	*N*=51	(%)	
Age (y)							
18-24	6	5.8	3	2.9	3	2.9	0.41
25-30	20	19.6	10	9.8	10	9.8	
31-39	54	52.9	27	26.49	27	26.49	
>40	22	21.56	11	10.78	11	10.78	
Marital status							
Ever married	77	75.49	41	40.2	36	35.29	0.25
Never married	25	24.51	10	9.8	15	14.71	
Education duration (y)							
Primary	8	7.84	3	2.94	5	4.9	0.09
Secondary	41	40.2	16	15.69	25	24.51	
Tertiary	53	51.96	32	31.37	21	20.59	
Profession							
Civil servant	23	22.55	12	11.76	11	10.78	0.37
Business	23	22.55	13	12.75	10	9.80	
Private	32	31.37	18	17.65	14	13.73	
Housewife	15	14.71	4	3.92	11	10.78	
Student	9	8.82	4	3.92	5	4.9	
Religion							
Christian	97	95.1	49	48.04	48	47.06	0.55
Muslim	5	4.9	2	1.96	3	2.94	

Statistical significance: *P*<0.05

### Obstetric and neonatal characteristics

Women in the IVF group had lesser lifetime pregnancies (*p* = 000) and delivered less than 2 children (*p* = 0.035). Consequently, they had less than 2 children alive (*p* = 0.019) (Table 2). The rate of multiple pregnancies was higher in the IVF group (*p* = 0000) and all the patients underwent antenatal care visits (Table 3).

**Table 2:** Obstetric characteristics of Patients

Characteristics	Total		IVF-ET		Controls		*P-Value*
	N=102	(%)	N=51	(%)	N=51	(%)	
Number of pregnancies							
0	27	26.47	19	18.63	8	7.84	0.000
1	56	54.90	29	28.43	27	26.47	
2-4	18	17.65	2	1.96	16	15.69	
5 and above	1	0.98	1	0.98	0	0	
Number of deliveries							
0	38	37.25	24	23.53	14	13.73	0.035
1	32	31.37	17	16.67	15	14.71	
2-4	29	28.43	10	9.80	19	18.63	
5 and above	3	2.94	0	0	3	2.94	
Number of children alive							
0	47	46.08	27	26.47	20	19.61	0.019
1	27	26.47	16	15.69	11	10.78	
2 and above	28	27.45	8	7.84	20	19.60	

Statistical significance: *P*<0.05

**Table 3:** Characteristics of pregnancies studied

Characteristics	Total		IVF-ET		Controls		*P-value*
	*N*=102	(%)	*N*=51	(%)	*N*=51	(%)	
Type of pregnancy							
Singleton	85	83.30	36	35.29	49	48.04	0.0000
Multiple	17	16.67	15	14.70	2	1.96	
ANC							
Yes	102	100.0	51	50.0	51	50.0	Ns
No	0	0.0	0	0.0	0	0.0	

*Ns* not significant, *ANC* Antenatal Care

### Delivery characteristics

Fewer women in the IVF group gave birth vaginally. This was attributed to the significantly higher cesarean delivery rate (58.8 %) in the IVF group compared to 27.5 % (*P* = 0.002) of the control group. Compared to the trend in spontaneous pregnancies, increase in preterm cesarean deliveries in the IVF group was insignificant. The birth weights and 5-min Apgar scores were similar in both groups (Table 4). The various indications for cesarean delivery are presented on Table 5. The leading factors of the increase in cesarean deliveries among IVF patients were advanced maternal age and IVF or precious baby. We did not induce labour or effect instrumental deliveries. Women with less than 2 children were more likely to have a cesarean delivery in the IVF group than in the controls (*P* = 0.009). Similarly, those with higher parities had significantly less cesarean deliveries in the IVF group than in the spontaneous pregnancy group (*P* = 0.005) (Table 6). There was a significantly higher cesarean section rate among women who did IVF out of Cameroon compared to those who went through the procedure in Cameroon (*P* = 0.006). The duration of infertility (*P* = 0.073) and the number of IVF trials (*P* = 0.059) did not influence the cesarean delivery rates to any significant degree (Table 7). In multiple regression analysis, women with IVF were at greater risk of cesarean section than spontaneous pregnancies (OR 4.096, 95 % CI 1.78–9.42) Table 8.

**Table 4:** Mode of delivery, gestational age, birth weights and Apgar scores at delivery

Characteristics	Total		IVF-ET		Controls		*P-value*
	*N*=102	(%)	*N*=51	(%)	*N*=51	(%)	
Mode of delivery							
Vaginal	58	56.86	21	20.59	37	36.27	0.002
Cesarean delivery	44	43.14	30	29.41	14	13.73	
Total	102	100.0	51	50.00	51	50.00	
Age of completed pregnancy (weeks)							
40±2 wks	84	82.35	36	35.29	48	47.06	0.53
<37 wks	18	17.65	15	14.71	3	2.94	
Total	102	100.0	51	50.00	51	50.00	
Birth weights (gm)							
<1000g	2	1.96	1	00.98	0	0.0	
1001-2000g	8	7.84	6	5.88	2	1.96	0.50
2001-2500g	15	14.70	8	7.84	7	6.86	
>2501g	77	75.49	36	35.29	42	41.18	
Total	102	100.0	51	50.00	51	50.00	
5 minute Apgar Score							
Less than 7	15	14.70	8	7.85	7	6.87	0.59
Greater than 7	87	85.30	43	42.15	44	43.13	
Total	102	100	51	50.00	51	50.00	

Statistical significance: *P*<0.05

< less than

> Greater than

**Table 5:** Indications for Cesarean delivery

Indication	Total		IVF-ET		Controls	
	*N*=44	(%)	*N*=30	(%)	*N*=14	(%)
Advanced maternal age at first pregnancy	12	27.27	10	22.73	2	4.55
IVF or precious pregnancy	8	18.18	8	18.18	0	0
Acute Fetal Distress	5	11.36	3	6.82	2	4.55
PMTCT (HIV)	1	2.27	0	0	1	2.27
Abruption Placenta	1	2.27	1	2.27	0	0
Others	17	38.64	8	18.18	9	20.45
Total	44	100	30	68.18	14	31.82

*PMTCT* Prevention of mother-to-child transmission, *HIV* Human immune-deficiency virus, % Percentage, *IVF* In-vitro fertilization

**Table 6:** Mode of delivery matched with number of children, gestational age, age and parity

Characteristic	Total		Vaginal Delivery		Cesarean Delivery		*P*
	N	(%)	N	(%)	N	(%)	
Number of children							
0	47	46.08	20	19.61	27	26.47	0.009
1	27	26.47	14	13.73	13	12.75	
≥2	28	27.45	24	23.52	4	3.92	
Total	102	100	58	56.86	44	43.14	
Gestational age(wk)							
40±2	57	55.88	47	46.08	10	9.80	0.53
≤36	45	44.12	37	36.27	8	7.85	
Total	102	100	84	82.35	18	17.65	
Age(yr)							
18-24	6 5	88	4	3.92	2	1.96	0.41
25-30	20	19.61	11	10.78	9	8.83	
31-39	54	52.94	33	32.35	21	20.59	
>40	22	21.57	9	8.82	13	12.75	
Total	102	100	57	55.87	45	44.13	
Parity							
0	38	37.25	16	15.69	22	21.57	0.005
1	32	31.37	15	14.71	17	16.67	
2	29	28.43	24	23.53	5	4.90	
≥5	3	2.94	2	1.96	1	0.98	
Total	102	100	57	55.88	45	44.12	

Statistical significance: *P*<0.05

**Table 7:** Mode of delivery based on where IVF was done, duration of infertility and number of IVF-ET trials

Characteristic	Total		Vaginal delivery		Caesarean delivery		P
	*N*	(%)	*N*	(%)	*N*	(%)	
IVF							0.006
Cameroon	32	62.75	16	31.37	16	31.37	
Abroad	19	37.25	6	11.76	13	25.49	
Total	51	100	22	43.14	29	56.86	
Duration of infertility (yr)							0.07
1	3	5.88	2	3.92	1	1.96	
2-5	30	58.82	13	25.49	17	33.33	
6-10	15	29.41	5	9.80	10	19.61	
>10	3	5.88	1	1.96	2	3.92	
Number of trials							0.06
1	16	31.37	7	13.72	9	17.65	
2	22	43.13	9	17.65	13	25.49	
3	10	19.61	4	7.84	6	11.76	
>4	3	5.88	1	1.96	2	3.92	
Total	51	100	21	41.18	30	58.82	

Statistical significance: *P*<0.05

**Table 8:** Odds Ratios [OR] and 95% Confidence Intervals [CI] of Deliveries

Variables	OR	95 %CI	*P*
Technique of conception			
(IVF-ET/Naturally)	4.10	1.78-9.42	0.0009
Age (y)			
(25–30 /18-24 )	1.64	0.24-11.08	0.614
(31-39/18-24 )	1.27	0.21-7.57	0.79
(>40 /18-24 )	2.89	0.43-19.28	0.27
Number of children			
One child versus no child)	0.66	0.26-1.71	0.40
(>2 children versus no child)	0.13	0.039-0.436	0.0009
Parity			
(primiparous versus nulliparous)	0.70	0.267-1.82	0.46
(≥2 deliveries versus nulliparous)	0.16	0.05-0.49	0.001
Duration of Infertility (y)			
(2–5 versus1y)	2.57	0.211-31.33	0.46
(6–10 versus1y)	5.00	0.348-71.90	0.24
IVF Center			
(Abroad versus Cameroon)	1.92	0.585-6.31	0.28
Number of IVF trials			
(2 trials versus1 trial)	1.36	0.37-5.07	0.65
(3 trials versus1trial)	1.17	0.23-5.81	0.85
(>4 trials versus 1 trial)	1.56	0.12-20.85	0.74
Gestational age at Delivery			
(Preterm versus Term)	1.37	0.49-3.81	0.55
Hospital			
(CDA versus DGH)	0.44	0.19- 1.05	0.0635

*OR* Odd’s ratio, *CI* confidence interval, *DGH* Douala General Hospital, *CDA* Clinique de L Aeroport

## Discussion

Women who conceive after IVF are usually older than those who conceive naturally; they are also often more primiparous, and have a poorer obstetric history [3]. These characteristics are all predictive of increased obstetric risk and adverse outcomes. A comparison with a control group is therefore mandatory if the prediction is to be confirmed or dismissed. In the present study, the obstetric outcomes of 51 IVF pregnancies were compared with those of 51 women who conceived naturally. All subjects were delivered in two hospitals: CDA and DGH, Cameroon. The socio-demographic characteristics were similar in the study and control groups.

Vaginal deliveries were significantly lower in the IVF group, and cesarean sections significantly higher. The global cesarean section rate in this study was 43.14 %: IVF 29.41 %, control group 13.73 %. This rate is similar to those reported previously [5, 6, 9, 10]. Women who conceived by IVF were 4 times more likely to have a cesarean delivery than those who conceived normally. Considering the comparatively low rates of antenatal complications in the control group, it seems reasonable to assume that the high rate of cesarean sections amongst IVF patients was at least in part a reflection of the equally high anxiety surrounding the management of these pregnancies. This assumption is supported by the fact that, amongst the indications for cesarean section, IVF or precious pregnancy was the next most frequent, immediately after advanced maternal age.

We used only maternal age and delivery dates to match the IVF group with that of spontaneous pregnancies; unlike Reubinof et al. who matched cases and controls with such other socio-demographic data as maternal age, parity, ethnic group, residence and delivery date [5].

Most of the IVF cases were women with advanced maternal ages; a fact which in itself could – and did – increase their obstetrical risk factors [11, 12]: the leading indication for cesarean section was advanced maternal age. Obesity and advanced maternal ages were seen to increase the risk of diabetes and cesarean delivery respectively. Among multiparas, this increase in obesity resulted either from excessive post-partum weight retention or then from weight gain between pregnancies. Obesity causes adverse pregnancy outcomes such as pre-eclampsia, gestational hypertension, gestational diabetes and cesarean delivery [13]. Several studies have confirmed that pregnancy is a trigger for excessive weight retention in many women. The Stockholm Pregnancy and Women’s Nutrition (SPAWN) Study carried out in Sweden [14, 15], followed up parous women 15 years after pregnancy and reported several factors that led to excessive weight gain, among which were a higher pre-pregnancy body mass index (BMI), higher gestational weight gain, more retained weight at 1 year post-partum, and a greater weight gain between 1-year and 15-year follow-up. The study also reported greater weight retention among parous women who had not breastfed and who had stopped smoking during pregnancy.

In terms of age distribution among study patients, 52 % were between 31 and 39, while 21.6 % were 40 and above. This corroborates the findings of Al-Turki who reported an average age of 37.38 ± 4.1 years [16] Many, 54.9 % of women in the study group had no children alive, as compared to 39.2 % in the control group. The chances of having multi-fetal pregnancies were 7.5 times higher in cases than in controls. Some studies have linked infertility and IVF treatment with multi-fetal gestations [7, 17], but others have reported only 12.7 % multi-fetal pregnancy rates [18].

There were more preterm deliveries in the IVF group than in the controls. Filicori et al. [19] and other studies attributed the high rates of preterm deliveries in IVF cases to the increased rates of multiple pregnancies after infertility treatments [20, 21]. On the contrary, Yang et al. [22] did not find any differences amongst the two groups.

IVF-assisted conception has been found to lead to negative outcomes such as preterm, low birth weight and perinatal/infant mortality [23]. Several studies have also reported adverse outcomes among women with delayed conception resulting from untreated infertility.

There is strong evidence that advanced maternal age contributes significantly to congenital malformations and later on in the offspring’s adolescence and adulthood, to an increased risk of cancer, neurologic disorders and cardiac diseases [24, 25]. Older paternal age has been associated with an increase in spontaneous abortions, preterm birth and congenital anomalies, with the highest risks when both partners are older [26–28]. Older maternal age requires more aggressive therapies to achieve a pregnancy including transferring more embryos.

Most patients in the IVF group in this study were married, had undergone tertiary level of education and were workers therefore could afford for IVF treatment though not significant. It is worth noting that the funding and regulatory framework for the provision of ART treatment varies considerably around the world and tends to be in line with the level of public and private responsibilities for purchasing healthcare. Public financing of ART ranges from virtually no subsidization in the USA and most developing countries including Cameroon to funding of a limited number of cycles based on female age in most European countries; to unrestricted reimbursement with co-payments in Australia [29]. The International Federation of Fertility Societies (IFFS) survey showed that roughly 50 % of countries had no reimbursement through national health services or private insurers in 2004 [30]; however, there was a higher proportion of countries with some level of subsidization than in the previous survey undertaken in 2002 [2, 29]. Those women who underwent IVF in a foreign country other than Cameroon were more likely to undergo a cesarean section thereby increasing the cost of obstetric care.

Among singleton pregnancies, assisted reproductive technology is associated with increased risks of preterm birth and low birth weight infants, which could explain the difference in Apgar scores reported in other studies [7, 31]. The birth weights and 5 min Apgar scores in our study were comparable between the cases and controls. IVF patients must be counselled about this risk before initiation of treatment.

The other variables studied did not influence much the cesarean delivery rate in our study: duration of infertility, the center where the IVF was performed, the number of IVF trials, the gestational age of pregnancy, and the hospital where the patient gave birth.

In our study we usually transferred 3 fresh embryos but recent studies have shown that the cumulative live birth rate (LBR) is as good as or better with single embryo transfer (SET) over 2 cycles than with two embryos transferred (DET) in 1 cycle, while greatly reducing the probability of a multiple birth [32–34]. There is need for our team to conform to current norms regarding number of embryos to be transferred.

Recent studies have also shown that women who conceived with infertility treatment were 2.95 times (95 % CI: 1.47–5.92) more likely to have planned cesarean deliveries. The increased risk for planned cesarean deliveries among singleton women who conceived with infertility treatment cannot be explained by older maternal age or higher number of morbidities during pregnancy. Counseling for women who conceive with infertility treatments may be needed to decrease unnecessary cesarean deliveries [35]. Furthermore, women with multifetal pregnancies are encouranged to try vaginal delivery [36, 37] or undergo multifetal pregnancy reduction (MFPR) [38–40].

### Study limitations

The limitation to this study was the inability of matching the two groups based on gestational age, parity and the type of pregnancy (singleton or multiple). We did not also study the difference in socioeconomic status between study participants who underwent IVF abroad compared to those who did it in Cameroon and the effect of weight gain or obesity on the participants. Our study population was small compared to similar studies with about 500 patients for study and longer study periods such as those of Adler-Levy et al. in Israel [11] and Shevell et al. in a multicenter study in the United States of America (USA) [12].

Furthermore, there was no communication between the two centres where the pregnant IVF women were followed-up or gave birth. Protocols amongst the two centres may have been different accounting for increased rate of cesarean births in one centre than another. Some IVF women started their pregnancy follow-up in other health facilities and only came to the two study centres to give birth. We may have also lost some IVF cases who did not return to any of the two study centres for ANC or pregnancy follow-up especially those who did the IVF out of Cameroon. We did not study separately the outcome of singleton and multifetal pregnancies to quantify the individual effect on cesarean section increase.

## Conclusions

The cesarean delivery rate was significantly higher in the cases than controls and there is a four times odds of having a cesarean delivery among those who conceived by IVF than controls. Patients who had delivered at least two times and those with over two children alive were less likely to have a cesarean delivery compared to those who had never delivered.

The leading indication for cesarean delivery was advanced maternal age in primigravida followed by IVF or precious pregnancy because of anxiety, over cautiousness and fear of birth trauma.

Further studies would examine the long-term neonatal outcomes beyond 5 min Apgar scores among IVF babies compared with matched controls and longer period of study beyond one year would be necessary.
